# Maine Veterinary Medical Association

**Published:** 1894-10

**Authors:** H. H. Choate

**Affiliations:** Secretary


### SOCIETY PROCEEDINGS.

### MAINE VETERINARY MEDICAL ASSOCIATION.

*October, 1894.*

A regular meeting of the Maine Veterinary Medical Association was held at the Elmwood Hotel, Waterville, October 10th, at 8 P. M. The President, Dr. Bailey, in the chair. The following members answered to the roll call: Drs. Bailey, Lord, Joly, Dwinal, Russell and Choate.

The minutes of the last meeting were read and accepted. The proposed amendment to the by-laws was adopted, as follows: It is proposed that Section 4, Article III—five members shall constitute a quorum for the transaction of business—shall be amended so as to read, three members shall constitute a quorum for the transaction of business.

The committee on professional fees was requested by the President to report at the next meeting.

Drs. W. D. Farnum, Rockland; Alfred L. Murch, Bangor; H. S. Usher, Hollis, Maine, were elected to membership in the Association.

Dr. Dwinal read a paper on Acute Cerebral Inflammations, as follows:

### ACUTE CEREBRAL INFLAMMATIONS, EMBRACING CEREBRITIS AND MENINGITIS.

DR. C. F. DWINAL, Bangor, Maine.

To comply with a request from the President, Dr. Bailey, that I should prepare a paper upon some subject to be chosen by me, I have selected Acute Cerebral Inflammations, embracing Cerebritis and Meningitis, which I will submit to you for your consideration.

I do not propose in this to speak much from experience, but to help to bring the disease before your mind. Acute cerebral inflammations are not so commonly met with in practice as many other diseases, and perhaps it was for this reason I selected this subject. Under this heading we have encephalitis, cerebritis or inflammation of the substance of the brain, and meningitis or inflammation of the coverings of the brain. The brain, the seat of animal life, is situated within the cranial cavity and covered or protected by three membranes, namely: dura mater, which lines the cranial cavity, pia mater, which covers the brain in all its convolutions, and the arachnoid, which is between these two. Inflammation is brought about in the brain and these membranes in several different ways, as the result of direct violence to the bones of the cranium or some disease of those bones, as the result of some specific fever, from the entrance into the system of some specific virus, as in cerebro-spinal fever, or from exposure to the rays of the sun. The most common cause, however, is from injuries to the cranial bones or disease of them, although we frequently see a sympathetic cerebral inflammation arising from some dietetic error.

Much time has been spent in endeavoring to discover some diagnostic symptoms by which to distinguish cerebral from meningeal inflammation, with some success; but we find that inflammation of the meninges quickly extends to the brain, and vice versa, therefore the distinction is not so desirable, only so at the outset.

Some of the diagnostic symptoms are these in meningitis: When the membranes are primarily affected we see the animal in a very excited condition, frightened at every object and avoiding the attendant in every way possible; he is startled at any noise or bright light penetrating the stall—in fact, spasms or convulsions, pain and delirium, are the general features of meningeal disease. Following these symptoms are diminution or loss of nervous functions.

In cerebral diseases we find the reverse of these symptoms, for from the outset or very early stages there is loss of one or more of the nervous functions, such as paralysis anæsthesia or loss of memory. The animal stands in a sleepy manner, with its head pressed against some firm object, and unmindful of anything going on about him.

The most common form of cerebral inflammation is due to some dietetic error, causing a sympathetic affection. This form may be distinguished from cerebral disease from other causes by one symptom in particular, that is the normal or even the sub-normal temperature, for in the former there is a rise in temperature.

Two cases which came under my observation will illustrate meningitis and sympathetic cerebritis.

The first, a case of acute meningitis, was in a black gelding, which I saw about seven o'clock in the evening. I was told that in the early afternoon he had been taken away to be clipped, and being a nervous animal had caused them some trouble, and in endeavoring to punish him had struck him with something, and had placed upon his head some kind of a controlling bridle, and had thrown him down.

When I arrived, he was in a stage of great excitement, so much so that they warned me about going into the stall, which was a roomy box, saying that he was crazy and would jump on me.

I finally got up to him, and putting my hand on him, found his head very warm, pulse quick, eyes staring and bloodshot, with pupils contracted, owing to his great anæsthesia. I was unable to take his temperature; there was much pain about the head; his appetite was very good, however, and continued so throughout this sickness.

In a few days this delirium changed to the comatose condition, and the animal stood in a corner, unmindful of anything; this, however, gradually wore away, and the animal made a good recovery.

Case number two, instance of sympathetic cerebral disturbance, was in a bay mare, which had been working constantly on a farm, had been fed meal in large quantities for a few days, and suddenly refused to eat and drink, and soon began to bore her head against the wall; eyes dull, and indifferent to everything, except occasionally she would thrash her head, thus wounding it greatly, and then remain quiet again.

Her eyes and lips were swollen very much when I saw her, pulse was hard, full and slow, temperature 101 degrees Fahrenheit, respiration stertorous.

With difficulty we got her from her position in the corner. By giving her a brisk cathartic and local applications to head, she very soon recovered. She had been affected this way once before, so I was told, but after this, having advised the owner not to feed any more meal, she has never been affected.

These two cases I think very well illustrate the two common diseases coming under the subject which I have chosen for my paper this evening.

Dr. Russell read a paper on Diagnosis of Tuberculosis, which was well discussed.

A motion was made and seconded that the Association tender the essayists a vote of thanks for the able manner in which they handled their subjects. Carried.

Drs. Lord and Choate were appointed to read papers at next meeting. The next meeting will be held at Augusta, January, 1895.

H. H. CHOATE, D. V. S., *Secretary.*

---

